# A cross-sectional exploratory study of rat sarcoma (Ras) activation in non-obese women with and without polycystic ovary syndrome

**DOI:** 10.17179/excli2025-9005

**Published:** 2026-01-09

**Authors:** Sara Anjum Niinuma, Haniya Habib, Ashleigh Suzu-Nishio Takemoto, Thozhukat Sathyapalan, Stephen L. Atkin, Alexandra E. Butler

**Affiliations:** 1 Royal College of Surgeons of Ireland, Adliya, Bahrain; 2 Academic Endocrinology, Diabetes and Metabolism, Hull York Medical School, Hull, UK

**Keywords:** Polycystic ovary syndrome, PCOS, cardiovascular risk, biomarkers, proteomics, rat sarcoma proteins, Ras, non-obese

## Abstract

Studies in obese polycystic ovary syndrome (PCOS) have shown growth factors that activate rat sarcoma (Ras) proteins, which regulate intracellular signaling pathways, differ in PCOS; however, it is difficult to account for obesity, insulin resistance, and systemic inflammation that are linked to many of the features found in PCOS. This study explores Ras signaling proteins and related growth factors in non-obese women with and without PCOS. Somascan proteomic analysis of circulating KRas, Ras GTPase-activating protein-1 (RASA1), and 45 growth factor-related proteins that signal through Ras was undertaken in a non-obese population of women with (n=44) and without (n=78) PCOS, groups matched for age and body mass index (BMI), without insulin resistance (HOMA-IR) or systemic inflammation (normal CRP; C-reactive protein). There was an increase in the free androgen index (FAI, p<0.0001) and anti-Müllerian hormone (AMH, p<0.0001) in PCOS. Cohen's *d* showed a moderate effect size for 3 proteins, of which Vascular endothelial growth factor-A (VEGFA) and EGFR were increased and EGFR1 was decreased in PCOS (all FDR p<0.05). EGFR and VEGF pathways interact closely and when EGFR signaling decreases, VEGFA may increase to maintain angiogenic balance, suggesting that in non-obese PCOS there may be a signal for compensatory angiogenesis in a dysfunctional endothelial environment.

See also the graphical abstract(Fig. 1).

## Introduction

Polycystic ovary syndrome (PCOS) is the most common endocrine disorder affecting 5-15 % of women of reproductive age worldwide, depending upon diagnostic criteria (Bozdag et al., 2016[7]; Salari et al., 2024[39]). It is characterized by a range of clinical features, including irregular menstrual cycles, anovulatory infertility, hyperandrogenism and hirsutism (Ehrmann, 2005[13]; Spritzer et al., 2022[44]). Beyond reproductive dysfunction, PCOS is also associated with an increased risk of metabolic comorbidities such as insulin resistance (IR), obesity, type 2 diabetes (T2D), metabolic syndrome and other cardiovascular disorders (Ehrmann, 2005[13]; Goodarzi et al., 2011[17]; Moran et al., 2015[29]; Zhu et al., 2019[55]). 

A well-established connection in PCOS pathophysiology is its strong association with both IR and obesity. Studies indicate that 38-88 % of women with PCOS are classified as overweight or obese, and the majority (50-90 %) exhibit IR (Barber et al., 2016[4], 2019[5]). Given that IR is exacerbated by obesity, it is proposed that increased adipose tissue dysfunction in obesity leads to chronic inflammation, altered adipokine secretion and lipotoxicity, thus further aggravating IR though, of note, this pathway is largely mediated via pro-inflammatory cytokines such as tumor necrosis factor alpha (TNFα) and interleukin 6 (IL6) (Chait and Den Hartigh, 2020[9]; Hammarstedt et al., 2018[18]; Kahn and Flier, 2000[21]). In turn, IR plays a critical role in PCOS pathophysiology. Hyperinsulinemia, a compensatory mechanism of IR, activates the insulin signaling MAPK (Mitogen-Activated Protein Kinase) pathway, which regulates cellular growth, differentiation and steroidogenesis (Santarpia et al., 2012[40]). Specifically, hyperinsulinemia stimulates ovarian theca cells to produce excess androgens, disrupting follicular maturation and contributing to anovulation and menstrual irregularities, hallmark clinical features of PCOS (Barber et al., 2019[5]; White et al., 1995[51]). The frequent co-occurrence of obesity and PCOS, alongside their shared link to IR, underscores the complex interplay of metabolic and endocrine dysfunction in the syndrome. 

However, while obesity and IR are central to PCOS pathology, a significant subset of women with PCOS do not present with obesity yet still experience menstrual irregularities, hyperandrogenism, hyperinsulinemia and metabolic disturbances (Carmina and Lobo, 2022[8]; Toprak et al., 2001[48]; Zhu et al., 2019[55]) This suggests that mechanisms beyond obesity-driven IR contribute to the syndrome. One potential target for further investigation is the insulin signaling MAPK pathway, specifically the Rat sarcoma (Ras) proteins, which serve as key regulators of cellular growth, differentiation and apoptosis through growth factor-mediated signaling (Simanshu et al., 2017[42]).

**Figure 2:**
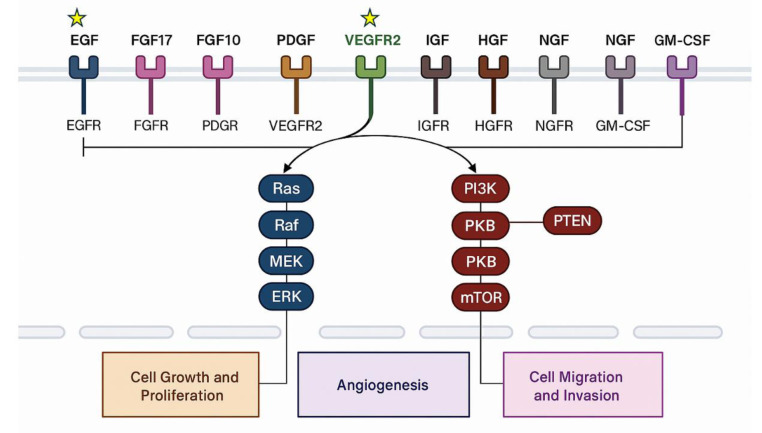
Illustration of growth factors and their activation of one of two intracellular pathways. Star symbols indicate dysregulated pathways in the current study. *Abbreviations:* EGF: Epidermal Growth Factor, EFGR: Epidermal Growth Factor Receptor, FGF: Fibroblast Growth Factor, FGFR: Fibroblast Growth Factor Receptor, PDGF: Platelet-derived Growth Factor, PDGFR: Platelet-derived Growth Factor Receptor; VEGF: Vascular Endothelial Growth Factor, VEGFR: Vascular Endothelial Growth Factor Receptor, IGF: Insulin and Insulin-like Growth Factor; IGFR: Insulin and Insulin-like Growth Factor receptor, HGF: Hepatocyte Growth Factor, HGFR: Hepatocyte Growth Factor Receptor, NGF: Nerve Growth Factor, NGFR: Nerve Growth Factor Receptor, GM-CSF: Granulocyte-Macrophage Colony-Stimulating Factor, GM-CSFR: Granulocyte-Macrophage Colony-Stimulating Factor Receptor, Ras: Rat Sarcoma Virus, RAF: Rapidly Accelerated Fibrosarcoma, MEK: Mitogen‑activated Protein Kinase Kinase, ERK: Extracellular Signal‑regulated Kinase, PIK3: Phosphoinositide 3-Kinase, PIP2: Phosphatidylinositol 4,5-bisphosphate, PTEN: Phosphatase and tensin homolog, pkB: Protein Kinase B, mTOR: Mammalian Target of Rapamycin

Previous research has provided evidence that circulating growth factors acting through Ras-mediated signaling pathways differ between individuals with PCOS and healthy controls at a BMI up to 29kg/m^2^ (Niinuma et al., 2025[31]) with epidermal growth factor receptor (EGFR), fibroblast growth factor 8 (FGF8), fibroblast growth factor receptor 1 (FGFR1), vascular endothelial growth factor D (VEGFD) insulin like growth factor 1 (IGF1) and its receptor (IGF1sR) differing, but when subsequently stratifying for HOMA-IR, only FGFR1, VEGFD, IGF1 and IGF1sR differed between groups. These growth factors interact with specific cell surface receptors to activate Ras signaling cascades, influencing key processes such as follicular development, insulin signaling and metabolic regulation (Apte et al., 2019[3]; Kobayashi et al., 2024[22]; Orian-Rousseau et al., 2007[32]; Ornitz and Itoh, 2015[33]; Pandey et al., 2023[35]; Reichardt, 2006[36]; Surve et al., 2021[47]; Werner, 2023[50]). Upon binding to transmembrane receptors, these circulating growth factors initiate one of two major intracellular signaling pathways, which may contribute to the underlying mechanisms of PCOS, as illustrated in Figure 2(Fig. 2). Notably, endothelial dysfunction proteins differ in obese, weight-matched women with PCOS and persisted after BMI was stratified (Niinuma et al., 2025[31]), highlighting the need to identify PCOS-related features that are independent of obesity in matched cohorts.

To address this gap, our study focused on non-obese (BMI 20-25 kg/m²), weight-matched women with and without PCOS, to investigate the role of Ras activation in the absence of obesity-driven metabolic dysfunction. We hypothesized that non-obese, insulin-sensitive and without overt systemic inflammation (normal CRP) weight-matched women with and without PCOS would not differ and would not show a difference in plasma growth factors that activate Ras.

## Materials and Methods

### Study design

In this exploratory cross-sectional study, plasma growth factors that activate Ras were determined in women with PCOS (*n*=44) and control women (*n*=78), recruited from the Hull in vitro fertilization (IVF) clinic (Cunningham et al., 2019[10]) and from a PCOS bio bank (Niinuma et al., 2025[31]); elements of both databases have been reported previously. Control and PCOS women were age- and BMI-matched. All procedures accorded with the ethical standards of the Yorkshire and The Humber NRES ethical committee, UK (ethics number 02/03/043) and Newcastle & North Tyneside Ethics Committee (reference number 10/H0906/17), which approved the studies. 29 PCOS women and 29 control women were recruited from the IVF clinics prior to hormonal treatment whilst the remainder of the PCOS women were recruited from the general endocrine clinics, and controls were recruited by advert. For a PCOS diagnosis, Rotterdam consensus criteria were employed: (1) biochemical (free androgen index (FAI) >4) and clinical (Ferriman-Gallwey score >8) hyperandrogenemia (2) amenorrhea or oligomenorrhea and (3) transvaginal ultrasound diagnosis of polycystic ovaries (Rotterdam ESHRE/ASRM-Sponsored PCOS consensus workshop group, 2004[38]). No other condition/ illness was present in the study participants, and all were medication-free (including no over-the-counter medications) for ≥9 months prior to enrolment. Testing to exclude the following endocrine conditions was undertaken: Cushing's disease, hyperprolactinemia, non-classical 21-hydroxylase deficiency or an androgen-secreting tumor (Legro et al., 2013[26]). Demographic and biochemical characteristics of the control and PCOS women are presented in Table 1(Tab. 1) (Reference in Table 1: Nandakumar et al., 2024[30]).

After overnight fasting, waist circumference, height (centimeters) and weight (kilograms) (to calculate body mass index (BMI) using the formula kg/m^2^) were measured per WHO guidelines (WHO, 2011[52]). Fasting bloods were centrifuged (3500 g x 15 minutes) within 5 minutes of venesection, aliquoted and stored (-80 °C). Anti-Müllerian hormone (AMH), sex hormone binding globulin (SHBG), insulin, C reactive protein (CRP) (DPC Immulite 200 analyser, Euro/DPC, Llanberis UK), and plasma glucose (for calculation of homeostasis model assessment-IR (HOMA-IR)) (Synchron LX20 analyser, Beckman-Coulter, High Wycombe, UK) were measured. FAI was calculated by dividing total testosterone by SHBG x100. Serum testosterone was determined by isotope-dilution liquid chromatography tandem mass spectrometry (LC-MS/MS) (Cunningham et al., 2019[10]).

Plasma proteins associated with Ras activation function were quantified by the Slow Off-rate Modified Aptamer (SOMA)-scan platform as described previously (Gold et al., 2010[16]; Suhre et al., 2017[46]). In brief, EDTA plasma samples were treated according to the following steps: 1) Analyte/primer beads binding-SOMAmers (consisting of a synthetic fluorophore-labeled SOMAmer bound via a photocleavable linker to a biotin moiety) underwent equilibration; 2) Analyte/SOMAmer complexes were immobilized using a streptavidin-substituted matrix. 3) UV light was used to cleave and thus analyte-SOMAmer complexes were released into solution; 4) subsequent immobilization of analyte-SOMAmer complexes via analyte-borne biotinylation onto a streptavidin matrix 5) Elution of complexes allowing release of SOMAmers that act as analyte quantification surrogates; 6) Hybridization to SOMAmer-complementary oligonucleotides to enable quantification. Standards were used for calibration as described previously (Kraemer et al., 2011[23]). Standardization and normalization of raw intensities, hybridization, median signal and calibration signal were undertaken (Kahal et al., 2020[20]; Kraemer et al., 2011[23]).

We used version 3.1 of the SOMAscan Assay. The proteins measured can be grouped into 3 main categories (growth factors, growth factor receptors and signaling pathway proteins as shown in Supplementary information, Supplementary Table 1) and included: GTPase kirsten rat sarcoma virus (KRAS), Ras GTPase-activating protein 1 (RASA1), Heparin-binding epidermal growth factor-like growth factor (HBEGF), Epidermal growth factor receptor variant III (EGFRvIII), Epidermal growth factor (EGF), Epidermal growth factor receptor (EGFR), Fibroblast growth factor 8 isoform A (FGF8A), Fibroblast growth factor 8 isoform B (FGF8B), Fibroblast growth factor receptor 1-4 (FGFR1, FGFR2, FGFR3, FGFR4), Fibroblast growth factor 2 (FGF2), Fibroblast growth factor 4-7 (FGF4, FGF5, FGF6, FGF7), Fibroblast growth factor 9-10 (FGF9, FGF10), Fibroblast growth factor 12 (FGF12), Fibroblast growth factor 16-20 (FGF16, FGF17, FGF18, FGF19, FGF20), Fibroblast growth factor 23 (FGF23), Platelet-derived growth factor C (PDGFC), Platelet-derived growth factor subunit A (PDGFA), Platelet-derived growth factor subunit B (PDGFB), Platelet-derived growth factor receptor beta (PDGFRB), Platelet-derived growth factor receptor alpha (PDGFRA), Vascular endothelial growth factor A (VEGFA), Vascular endothelial growth factor A isoform 121 (VEGFA121), Vascular endothelial growth factor C (VEGFC), Vascular endothelial growth factor D (VEGFD), Vascular endothelial growth factor receptor 2 (VEGFsR2), Vascular endothelial growth factor receptor 3 (VEGFsR3), Insulin (INS), Insulin-like growth factor I (IGF1), Macrophage colony-stimulating factor 1 (CSF1), Macrophage colony-stimulating factor 1 receptor (CSF1R), Granulocyte-macrophage colony-stimulating factor (CSF2), Granulocyte colony-stimulating factor (CSF3), Granulocyte colony-stimulating factor receptor (CSF3R), Insulin-like growth factor 1 receptor (IGF1R), Cation-independent mannose-6-phosphate receptor (IGF2R), Hepatocyte growth factor (HGF), beta-nerve growth factor (NGF). Calibration was based on standards as previously described (Niinuma et al., 2025[31]).

### Statistics

Whilst this was an exploratory study, a power analysis was undertaken based on the change of FGFR1 reported between PCOS and controls (Niinuma et al., 2025[31]): for an alpha of 0.05 with 80 % power and an effect size of 0.7, a minimum of 26 subjects per arm were required. Descriptive statistics were used to compute the mean ± SD and the median (IQR) for the continuous variables. Student t-test or Wilcoxon rank sum test was used to assess the statistical differences of the continuous variables across the groups. Cohen's d analysis was undertaken for effect size, and the False Discovery Rate (FDR) was applied for multiple testing. All tests were two-tailed and a p-value <0.05 was considered significant. All analyses were performed using R Bioconductor packages, SPSS v 2 6.0 and Graphpad Prism version 10.0.0 (San Diego, CA, USA).

## Results

Demographic, hormonal and metabolic data for the 44 PCOS subjects and 78 controls are shown in Table 1(Tab. 1). The two cohorts were weight and age-matched and had neither IR (as adjudged by HOMA-IR) nor evidence of increased systemic inflammation, as CRP (mg L^-1^) was not elevated (normal range 0-3 mg L^-1^) and did not differ between the groups; subjects with PCOS did have hyperandrogenemia and elevated AMH (both p<0.0001 versus controls).

### Endothelial-associated protein levels

The results of the growth factor-related proteins that activate Ras intracellular signaling are shown in Table 2(Tab. 2) for PCOS and their respective control subjects for those with moderate and small effect sizes, whilst those with a negligible effect size are shown in Supplementary informaiton, Supplementary Table 2. Cohen's *d* showed a moderate effect size for 3 proteins, of which VEGFA and EGFR were increased (FDR p<0.05), whilst EGFR1 was decreased in PCOS (FDR p<0.05).

## Discussion

The non-obese cohort of PCOS patients recruited for this study was matched to controls for BMI, IR and CRP. We hypothesized that, with these parameters being normal, there would be no difference in growth factor-related protein markers signaling through Ras proteins. The results here showed that VEGFA and EGFR were increased, whilst EGFR1 was decreased in PCOS after correcting for multiple comparisons. EGFR, EGFR1 and VEGFA are tightly connected in endothelial, metabolic and ovarian signaling pathways (Lee et al., 2025[25]). The elevation in soluble EGFR may be explained by increased ectodomain shedding mediated by ADAM10/17 metalloproteinases, which are strongly activated by inflammatory cytokines, hyperinsulinemia and oxidative stress found in PCOS, obesity and insulin resistant states (Blobel, 2005[6]; Malaguarnera et al., 2025[28]). EGFR shedding may raise soluble EGFR levels while reducing membrane-bound EGFR1, that may be the explanation for the findings reported here. Similar dissociation between circulating EGFR and tissue EGFR1 has been reported in metabolic syndrome, early diabetes and endothelial injury models (Shraim et al., 2021[41]).

In comparison with our previous study, where BMI was stratified up to 29 kg/m^2^ (Niinuma et al., 2025[31]), EGFR differed in both studies; fibroblast growth factor 8 (FGF8), FGFR1, IGF1 differed in the previous study and here they were either significantly different (IGF1, p=0.05) or showed a trend (FGF8, FGFR1) towards significance (0.06 and 0.07, respectively), but none remained significant after FDR testing. VEGFD, and IGF1sR, that differed previously (18), could not be compared in this study as the SOMA panel used for the samples in this study did not contain either. The major difference between the studies was that here the BMI was 20-25 kg/m^2^ in comparison to a BMI of up to 29 kg/m^2 ^(18), and the differences seen are likely due to BMI. In addition, in the previously reported study, all the PCOS patients were phenotype A (irregular menses with hyperandrogenism and polycystic ovaries on ultrasound). In this study, 15 of the PCOS patients were phenotype A, whilst the remainder were a mixture of phenotype B or C: phenotype B (irregular menses with hyperandrogenism); phenotype C (irregular menses and polycystic ovaries on transvaginal scanning). There were too few subjects, and less than the power analysis would allow, to perform a subgroup analysis.

VEGFA was elevated in PCOS patients in comparison to controls as has previously been reported (Agrawal et al., 1998[2], 2002[1]; Dambala et al., 2017[12], 2019[11]). VEGF is a pro-angiogenic factor whose increase can contribute to hyperplasia, hypervascularity and the gradual development of endothelial dysfunction, key features of PCOS (Ferrara et al., 2003[14]). However, age and PCOS phenotype may affect these growth factors; a study investigating adolescent PCOS patients found no significant differences in VEGF levels between cases and controls, suggesting that VEGF expression may vary depending on the development of PCOS and its subpopulations (Skrzynska et al., 2024[43]). VEGFA is expressed in granulosa and theca cells from early stages and rises as follicles grow, promoting angiogenesis in the thecal vasculature for the enlarging follicle, and further increases as a dominant follicle develops (Fraser, 2006[15]; Jankowska-Ziemak et al., 2025[19]). In PCOS, VEGFA rises not only as an endothelial response but also due to ovarian overproduction: hyperinsulinemia, elevated LH, and local oxidative stress in granulosa and theca cells stimulate VEGFA transcription, contributing to stromal hypervascularity and abnormal folliculogenesis (Li et al., 2020[27]). Clinically, VEFGA is thought to contribute to the development of ovarian hyperstimulation syndrome during controlled ovarian stimulation (Ferrara et al., 2003[14]). Ovarian cells express vascular endothelial growth factor receptor 1 (VEGFR1/FLT1) and vascular endothelial growth factor receptor 2 (VEGFR2/KDR) (Ortega et al., 2015[34]), with VEGFR2 meditating most of the mitogenic/permeability effects of VEFGA. In this study, the increase of VEFGA was highly significant but in real terms there was only a 1.2-fold increase that, on its own, is unlikely to be clinically significant but, in combination with other factors, may have clinical relevance.

The present study showed significantly increased EGFR and reduced EGFR1 levels in women with PCOS. The EGFR axis is essential for normal folliculogenesis, granulosa cell proliferation, and the coordination of LH-mediated oocyte maturation. Altered expression of EGFR family signaling components has been associated with defective ovulatory responses in both human and experimental models (Zhang et al., 2022[54]). 

In healthy follicles, activation of EGF-family ligands triggers the EGFR-extracellular signal-related protein kinases 1 and 2 (ERK1/2) signaling cascade required for cumulus-oocyte complex expansion and luteinizing hormone (LH)-induced resumption of meiosis. Decreased EGFR or reduced phosphorylation of EGFR disrupts these downstream pathways, leading to insufficient mitogen-activated protein kinase (MAPK) activation and impaired oocyte maturation (Richani and Gilchrist, 2018[37]). A reduction in EGFR signaling may reflect in a functional deficiency in peri-ovulatory signaling events necessary for follicular progression and ovulation (Sugimura et al., 2018[45]).

Impaired EGFR function has also been linked to abnormal follicular angiogenesis. EGFR-mediated pathways normally cross-regulate angiogenic factors such as VEGFA; when EGFR signaling is reduced, compensatory upregulation of VEGFA may occur, that may be the mechanism seen here, and may suggest a decoupling of angiogenic and growth-factors within the PCOS ovary. Overall, the increased EGFR and decreased EGFR1 in PCOS may indicate an increase in angiogenic drive while EGF-mediated follicular maturation signaling declines, resulting in suboptimal oocyte development (Yang et al., 2016[53]). 

These findings suggest that growth factor-related proteins involved in Ras signaling differ among non-obese women with PCOS. This suggests that differences in Ras-mediated signaling specifically for VEGFA, EGF and EGFR may be inherent in PCOS, whilst the other Ras-mediated signaling growth factors observed in prior studies may be primarily driven by obesity, chronic inflammation or IR, or a combination of these parameters, rather than PCOS itself. Hypothetically, even a modest increase in weight, above what is considered normal, could lead to more pronounced Ras activation in women with PCOS compared to their non-PCOS counterparts at the same weight. 

From a translational perspective, EGFR-pathway dysregulation may represent a potential therapeutic target. Restoration of EGF-family signaling has been suggested as a potential strategy to improve oocyte competence in PCOS undergoing assisted reproduction (Vitale and Dolmans, 2024[49]).

Strengths of this study include that the cohort was a well-defined non-obese non-IR Caucasian PCOS population closely matched to controls, allowing comparison of a panel of growth factor-related protein markers signaling through Ras, without the bias of BMI, IR or chronic inflammation. Limitations include the small population size; however, the power analysis (though based upon FGFR1 changes between PCOS and controls) suggested that the study was adequately powered to reveal differences, but the findings should be treated with caution as exploratory, with further validation studies in larger cohorts being necessary. That the study was performed on an exclusively Caucasian population of non-obese women many of who were attending an IVF clinic, and who were thus potentially subfertile, may also be seen as a limitation and would need to be repeated in other ethnic groups, other BMI strata, and in women known not to be subfertile, are needed to confirm these findings. The use of CRP as the only measure of systemic inflammation is a limitation, as other more sensitive inflammatory markers may have shown elevations despite a CRP in the normal range. The use of HOMA-IR as the only measure of insulin resistance is a limitation, as this is an indirect measure and lacks the sensitivity of a euglycemic clamp, though multiple studies have shown a strong, statistically significant correlation between HOMA and clamp measurements (Lansang et al., 2001[24]). The cross-sectional study design can be viewed as a limitation, as temporal relationships between VEGFA, EGF and EGFR cannot be determined, nor can changes in these proteins be a cause or effect of the PCOS condition. 

## Conclusion

The EGFR and VEGF pathways interact closely and when EGFR signaling decreases, VEGFA may increase to maintain angiogenic balance, suggesting that in non-obese PCOS there may be a signal for compensatory angiogenesis in a dysfunctional endothelial environment. 

## Notes

Sara Anjum Niinuma, Haniya Habib, and Ashleigh Suzu-Nishio Takemoto contributed equally as first author.

## Declaration

### Ethics approval and consent to participate

All procedures performed in studies involving human participants were in accordance with the ethical standards of the Yorkshire and The Humber NRES ethical committee, UK (ethics number 02/03/043), which provided approval for the study, and with the 1964 Helsinki declaration and its later amendments or comparable ethical standards.

### Informed consent statement

Informed consent was obtained from all subjects involved in the study.

### Consent for publication

All authors gave their consent for publication.

### Availability of data and materials

All the data for this study will be made available upon reasonable request to the corresponding author.

### Conflict of interest

No authors have any conflict of interest or competing interests to declare.

### Funding

No funding was received to perform this study.

#### Author contribution statement

SAN, HH and AST wrote and edited the manuscript and contributed to visualization. AEB analyzed the data and wrote/edited the manuscript. TS supervised clinical studies and edited the manuscript. SLA contributed to study design, data interpretation and the writing/editing of the manuscript. All authors reviewed and approved the final version of the manuscript. Alexandra E Butler is the guarantor of this work.

#### Artificial Intelligence (AI) - assisted technology

Artificial intelligence was not used in the preparation of this manuscript.

## Supplementary Material

Supplementary information

## Figures and Tables

**Table 1:**
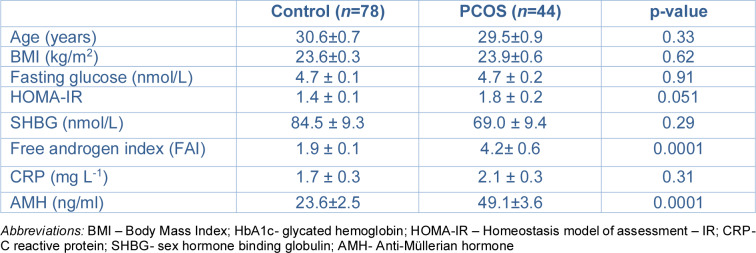
Demographics, baseline, hormonal and metabolic parameters of the PCOS subjects and controls (mean ± SEM) (Nandakumar et al., 2024)

**Table 2:**
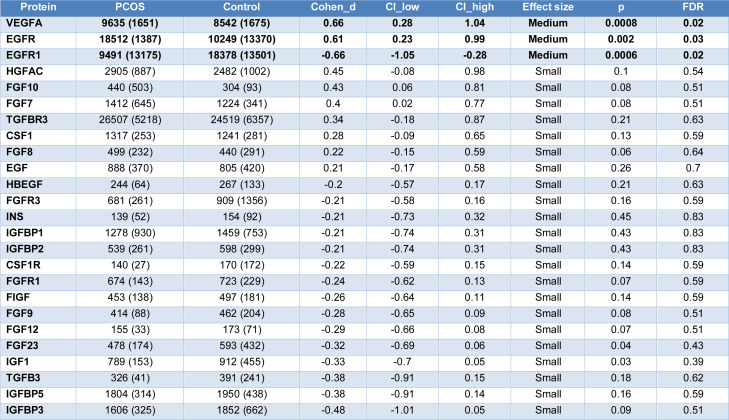
Proteins associated with endothelial dysfunction in non-obese Polycystic ovary syndrome (PCOS n=44) versus their non-obese BMI-matched controls (n=78). All proteins had a Cohens *d* medium effect size. Data presented as Mean ± 1 Standard Deviation of Relative Fluorescent Units (RFU)

**Figure 1:**
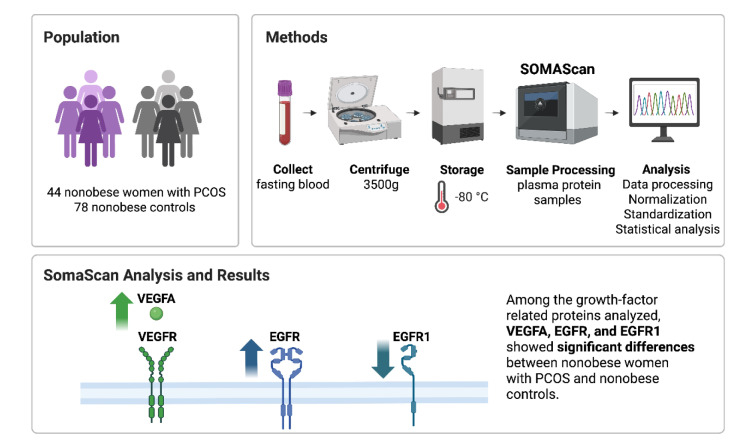
Graphical abstract
