# Supplementary material for: A cross-sectional exploratory study of rat sarcoma (Ras) activation in non-obese women with and without polycystic ovary syndrome
Source: EXCLI J. 2026 Jan 9;25:174–85. doi: 10.17179/excli2025-9005 (PMC12901957; doi:10.17179/excli2025-9005)
Supplement: Supplementary information [file EXCLI-25-174-s-001.pdf]

EXCLI Journal 2026;25:174-185 – ISSN 1611-2156
Received: October 08, 2025, accepted: December 01, 2025, published: January 09, 2026

Supplementary information to:

Original article:

A CROSS-SECTIONAL EXPLORATORY STUDY OF RAT SARCOMA (RAS) ACTIVATION IN NON-OBESE WOMEN WITH AND WITHOUT POLYCYSTIC OVARY SYNDROME

Sara Anjum Niinuma[1#], Haniya Habib[1#], Ashleigh Suzu-Nishio Takemoto[1#], Thozhukat Sathyapalan[2], Stephen L. Atkin[1], Alexandra E. Butler[1]

[1] Royal College of Surgeons of Ireland, Adliya, Bahrain
[2] Academic Endocrinology, Diabetes and Metabolism, Hull York Medical School, Hull, UK

[#] joint first authors

* **Corresponding author:** Alexandra E. Butler, Research Department, Royal College of Surgeons of Ireland, Bahrain, Adliya, Bahrain.
Phone: +97366760313, E-mails: aeb91011@gmail.com; abutler@rcsi.com

https://dx.doi.org/10.17179/excli2025-9005

This is an Open Access article distributed under the terms of the Creative Commons Attribution License (https://creativecommons.org/licenses/by/4.0/).

EXCLI Journal 2026;25:174-185 – ISSN 1611-2156
Received: October 08, 2025, accepted: December 01, 2025, published: January 09, 2026

Supplementary Table 1: Major classifications of the proteins included in this analysis. The 3 major classifications are growth factors (ligands), growth factor receptors and signaling pathway proteins.

**Growth Factors (Ligands)**	
EGF	Epidermal Growth Factor
FGF	Fibroblast Growth Factor
PDGF	Platelet-derived Growth Factor
VEGF	Vascular Endothelial Growth Factor
IGF	Insulin and Insulin-like Growth Factor
HGF	Hepatocyte Growth Factor
NGF	Nerve Growth Factor
GM-CSF	Granulocyte-Macrophage Colony-Stimulating Factor
**Growth Factor Receptors**	
EFGR	Epidermal Growth Factor Receptor
FGFR	Platelet-derived Growth Factor Receptor
PDGFR	Platelet-derived Growth Factor Receptor
VEGFR	Vascular Endothelial Growth Factor Receptor
IGFR	Insulin and Insulin-like Growth Factor receptor
HGFR	Hepatocyte Growth Factor Receptor
NGFR	Nerve Growth Factor Receptor
GM-CSFR	Granulocyte-Macrophage Colony-Stimulating Factor Receptor
**Signaling Pathway Proteins**	
Ras	Rat Sarcoma Virus
RAF	Rapidly Accelerated Fibrosarcoma
MEK	Mitogen-activated Protein Kinase Kinase
ERK	Extracellular Signal-regulated Kinase
PIK3	Phosphoinositide 3-Kinase
PIP2	Phosphatidylinositol 4,5-bisphosphate
PTEN	Phosphatase and tensin homolog
pkB	Protein Kinase B
Protein Kinase B	Mammalian Target of Rapamycin

EXCLI Journal 2026;25:XX-XX – ISSN 1611-2156
Received: January 24, 2025, accepted: April 26, 2026, published: XX, 2026

Supplementary Table 2: Proteins associated with endothelial dysfunction in non-obese Polycystic ovary syndrome (PCOS n=44) versus their non-obese BMI-matched controls (n=78). All proteins had a Cohens *d*, small effect size. Data presented as Mean ± 1 Standard Deviation of Relative Fluorescent Units (RFU)

Protein	PCOS_mean_(SD)	CTRL_mean_(SD)	Cohen's_d	CI_low	CI_high	Effect_Size	p	FDR
**RAC3**	4654 (2618)	4241 (1656)	0.19	-0.34	0.71	Negligible	0.48	0.83
**RAC1**	16704 (19544)	13704 (11167)	0.19	-0.34	0.71	Negligible	0.48	0.83
**VEGFC**	1068 (392)	1023 (254)	0.15	-0.23	0.52	Negligible	0.49	0.83
**TGFBI**	28586 (6470)	27744 (6035)	0.13	-0.39	0.66	Negligible	0.62	0.87
**PDGFRB**	4610 (2054)	4274 (2775)	0.13	-0.24	0.5	Negligible	0.45	0.83
**FGF19**	6312 (4714)	5800 (3747)	0.12	-0.25	0.5	Negligible	0.54	0.86
**KDR**	7972 (1674)	7796 (1711)	0.1	-0.27	0.48	Negligible	0.58	0.86
**VEGFA.1**	648 (296)	622 (280)	0.09	-0.28	0.46	Negligible	0.64	0.88
**TAB1**	1327 (2209)	1185 (726)	0.08	-0.44	0.61	Negligible	0.75	0.92
**IGF1R**	5745 (1569)	5630 (1325)	0.08	-0.29	0.45	Negligible	0.68	0.91
**IGFBP6**	464 (78)	458 (115)	0.07	-0.45	0.59	Negligible	0.8	0.95
**FGF2**	4540 (13419)	3767 (9582)	0.07	-0.3	0.44	Negligible	0.74	0.92
**IGFBP7**	34915 (8270)	34411 (10529)	0.05	-0.47	0.58	Negligible	0.84	0.95
**IGFBP4**	9999 (2224)	9890 (2992)	0.04	-0.48	0.57	Negligible	0.88	0.96
**FGF17**	172 (39)	172 (28)	0.01	-0.36	0.38	Negligible	0.97	0.99
**CSF3R**	1164 (771)	1162 (862)	0	-0.37	0.37	Negligible	0.99	0.99
**PDGFB**	30529 (25071)	30488 (24116)	0	-0.37	0.37	Negligible	0.99	0.99
**RASA1**	791 (659)	801 (910)	-0.01	-0.38	0.36	Negligible	0.95	0.99
**FLT4**	7750 (1997)	7795 (2154)	-0.02	-0.39	0.35	Negligible	0.91	0.98
**TGFB1**	1232 (1274)	1259 (841)	-0.03	-0.55	0.5	Negligible	0.92	0.98
**FGF20**	305 (58)	308 (104)	-0.03	-0.4	0.34	Negligible	0.84	0.95
**IGF2R**	30801 (8118)	31092 (8880)	-0.03	-0.41	0.34	Negligible	0.86	0.95
**FGF16**	1603 (811)	1631 (794)	-0.03	-0.41	0.34	Negligible	0.86	0.95
**PDGFA**	12028 (7479)	12445 (7604)	-0.06	-0.43	0.32	Negligible	0.77	0.93

EXCLI Journal 2026;25:XX-XX – ISSN 1611-2156
Received: January 24, 2025, accepted: April 26, 2026, published: XX, 2026

Protein	PCOS_mean_(SD)	CTRL_mean_(SD)	Cohen's_d	CI_low	CI_high	Effect_Size	p	FDR
**FGF18**	258 (64)	263 (68)	-0.07	-0.44	0.3	Negligible	0.71	0.92
**HGF**	1644 (436)	1699 (663)	-0.09	-0.46	0.28	Negligible	0.59	0.86
**CSF2**	235 (100)	250 (169)	-0.1	-0.47	0.28	Negligible	0.56	0.86
**PROK1**	137 (38)	143 (82)	-0.1	-0.62	0.43	Negligible	0.72	0.92
**PDGFRA**	1160 (528)	1386 (2658)	-0.11	-0.48	0.27	Negligible	0.47	0.83
**FGFR4**	1301 (1989)	1480 (1323)	-0.11	-0.48	0.26	Negligible	0.6	0.86
**FGF6**	275 (91)	297 (218)	-0.12	-0.49	0.25	Negligible	0.45	0.83
**FGF5**	701 (766)	1129 (4434)	-0.12	-0.49	0.25	Negligible	0.41	0.83
**TGFB2**	190 (75)	207 (189)	-0.12	-0.65	0.4	Negligible	0.66	0.89
**CSF3**	1309 (948)	1724 (3857)	-0.13	-0.5	0.24	Negligible	0.38	0.83
**FGFR2**	261 (160)	287 (190)	-0.15	-0.52	0.22	Negligible	0.42	0.83
**KRAS**	1057 (218)	1438 (3225)	-0.15	-0.52	0.22	Negligible	0.31	0.76
**NGF**	733 (563)	875 (1052)	-0.16	-0.53	0.21	Negligible	0.34	0.8
**PDGFC**	406 (294)	503 (677)	-0.17	-0.54	0.2	Negligible	0.28	0.72
**TGFBR2**	773 (299)	858 (605)	-0.18	-0.71	0.35	Negligible	0.51	0.84
**FGF4**	171 (43)	204 (220)	-0.19	-0.56	0.18	Negligible	0.2	0.63
**FGF8**	640 (153)	682 (234)	-0.2	-0.57	0.17	Negligible	0.25	0.68
